# Green-Stick Fracture of the Clavicle, with Remarks

**Published:** 1874-11

**Authors:** Jno. N. Upshur

**Affiliations:** Richmond, Virginia


### GREEN-STICK FRACTURE OF THE CLAVICLE—WITH REMARKS.

BY JNO. N. UPSHUR, M.D., OF RICHMOND, VIRGINIA.

I was called on the 15th of April, 1874, to see A. B., colored, æt 20 months, a very robust, healthy child, who had fallen down six stair-steps, striking on the left shoulder. Examination revealed a green-stick fracture of the clavicle about one inch from its acromial articulation. The diagnosis was easy; inspection showed less prominence just to the inside of the shoulder than on the uninjured side; the arm was drawn inwards, and drooped somewhat downwards, with more or less loss of the power of motion. The fracture could be felt beneath the skin, the concavity anteriorly being increased, and the splintered fibres on the convexity, at the point of injury, very perceptible. Handling caused pain. Chloroform was given, the fracture reduced, and Sayre's adhesive plaster apparatus applied to keep the limb at rest. In three weeks the bone was perfectly restored without deformity.

*Remarks.*—I have examined several authorities, but find nothing said about this particular fracture, except in Mr. T. Holme's work on "The Surgical Diseases of Infancy and Childhood." He gives a plate of a specimen of this fracture from the Museum of Saint George's Hospital, occuring in a child five years old, the fracture being at the same point as that described above. Mr. H. says that the "diagnosis is easy," and gives as the symptoms, "change in the axis of the limb, with pain and inability to move it, and a lump over the seat of injury." He says further that "the injury is perhaps not infrequent, but that all of the cases that he has seen and diagnosed occurred in the bones of the forearm." But Guersaut says that fracture of the clavicle is only more frequent than that of the jaw and ribs (*Surgical Diseases of Infants and Children, p.* 33. Translated by Richard J. Dunglison, M.D). Mr. Erichsen merely refers to its occasional occurrence, and says that this fracture of the clavicle is comparatively rare (*Ashurst's Ed.*).

I would highly commend the apparatus of Dr. Sayre mentioned above. It is simple in application, and answers all of the indications better than any other known to me.

